# Economic and livestock health impacts of birds on dairies: Evidence from a survey of Washington dairy operators

**DOI:** 10.1371/journal.pone.0222398

**Published:** 2019-09-19

**Authors:** Julie L. Elser, Amber L. Adams Progar, Karen M. M. Steensma, Tyler P. Caskin, Susan R. Kerr, Stephanie A. Shwiff

**Affiliations:** 1 National Wildlife Research Center, USDA/APHIS/WS, Fort Collins, CO, United States of America; 2 Department of Animal Sciences, Washington State University, Pullman, WA, United States of America; 3 Department of Biology, Trinity Western University, Langley, BC V2Y 1Y1, Canada; Universidade Federal de Mato Grosso do Sul, BRAZIL

## Abstract

The survey described in this research paper aimed to investigate the economic and health impacts of birds on dairies. Birds are common pests on dairies, consuming and contaminating feed intended for cattle. As a result, dairy operators experience increased feed costs and increased pathogen and disease risk. We surveyed dairy operators attending the 2017 Washington Dairy Conference to examine the impact of birds on dairies in Washington State. Dairy operators reported feed losses valued at $55 per cow resulting in annual losses totaling $5.5 million in the Western region of the state and $9.2 million in the Eastern region of the state. Shooting was the most commonly used bird management method and European starlings (*Sternus vulgaris)* were the most frequently implicated species statewide. Bird abundance greater than 10,000 birds per day was associated with larger herd size and with self-reported presence of Johne’s disease and *Salmonella*.

## Introduction

The pest bird problem on dairies in the Pacific Northwest (PNW), especially regarding invasive European starlings (*Sternus vulgaris)*, has been compounded with changes in land use and agricultural practices over the past several decades. In the PNW, dairies have become fewer in number but herd sizes have grown, such that the total number of dairy cows has remained relatively constant. This intensification has led to increased use of open commodity sheds with high-energy feeds such as grains and cottonseed, piled high for ease of access by loaders. Similarly, tarped, open-face bunker silos for corn silage and haylage have largely displaced enclosed, upright silos [1]. Unfortunately, these newer feed storage practices allow better access for foraging birds.

In other areas of the United States, the impact of birds on dairies has been linked to significant economic damages and disease transmission concerns [2–4]. Bird depredation of cattle feed may result in nutritional depletion of feed, which may impact milk production [5]. Depenbusch et al. [6] reported increases in daily production costs of $0.92 per animal at a Kansas feedlot due to feed consumption by starlings. A survey of commercial dairy operators in Pennsylvania, New York, and Wisconsin suggests dairies reporting 10,000 or more birds per day lost $64,000 of feed annually and were more likely to self-report *Salmonella* spp. and Johne’s disease [3]. However, that survey did not reveal an effect of bird presence on milk production.

Birds have been implicated in the transmission of pathogens due to their close contact with animals at livestock facilities. Birds may contaminate feed and water and/or transport pathogens between livestock facilities. Presence of high density bird flocks at livestock facilities creates conditions ideal for the transmission of pathogens between birds and cattle, and from birds to other birds [7]. Pigeons have been reported to acquire and recirculate pathogens including *Salmonella enterica* on dairies and to vector pathogens among multiple livestock facilities [8, 9]. Starlings may contribute to pathogen transmission by physically moving cattle feces, which may contain *Salmonella* spp., *E*. *coli*, and other pathogens, into feed and water troughs, thereby disseminating pathogens throughout livestock facilities [10]. Controlling starling presence on cattle feedlots was found to reduce *S*. *enterica* contamination in feed bunks and water troughs, although it had no effect on the prevalence of *S*. *enterica* in the cattle [11].

A comprehensive assessment of the impacts of birds on dairies in the PNW is not currently available. This survey of dairy operators in Washington State aimed to ascertain the current extent of bird damage in that region, including pathogen presence, bird management efforts, and economic impacts. The direct costs of bird presence on dairies were calculated and applied in a regional economic model to estimate the overall economic impact of birds to the state of Washington.

## Materials and methods

### Survey instrument and implementation

A survey was implemented by Washington State University’s Pest Bird Management group at the annual Washington Dairy Conference in November 2017, following the approach of Werner et al. [12]. The Washington State University Institutional Review Board reviewed all survey materials and categorized this research as exempt. The survey targeted commercial dairy operations in Washington State and consisted of 23 questions soliciting information including dairy demographics, herd health, production, expenses, and experiences with pest birds and bird management efforts. Most questions asked dairy operators to report data and experiences from 2016. Organizers estimated just under 100 dairy farms were represented at this conference (Washington State Dairy Federation, personal comm.). Surveys were distributed from WSU’s Pest Bird Management booth throughout the conference and on all tables at lunch on the first day of the conference. Each survey included a cover letter explaining the purpose of the survey, participation was voluntary, and responses would be confidential (the survey did not ask for names or addresses of respondents).

### Survey questions and analysis

#### Bird abundance and species identification

Dairy operators were asked whether birds were present in the facilities where they house milk cows. They were then asked to estimate the number of birds present on their operation per day at peak numbers. Respondents were given five abundance categories to choose from: under 1,000; 1,000 to 10,000; 10,001 to 25,000; 25,001 to 50,000; and over 50,000 birds. Carlson et al.[10, 11] established that operators can reliably estimate flock sizes within similar abundance ranges. Operators were asked to identify and rank bird species observed consuming feed and, in a separate question, identify and rank the species that caused the most damage to their overall operation. Ranks were from most common/damage (1) to least common/damage (6). Pictures of six bird species commonly found on livestock facilities were provided to help respondents identify species correctly: European starling, redwing blackbird (*Agelaius phoeniceus*), house sparrow (*Passer domesticus*), rock pigeon (*Columba livia*), American crow (*Corvus brachyrhynchos*), and brown-headed cowbird (*Molothrus ater*). Respondents also had the option to write in and rank other species. We used a scoring system in which the species ranked first by a respondent was assigned three points, second ranked two points, and third ranked one point. These points were aggregated to arrive at a single score for each species.

We performed regression analyses to determine relationships between bird abundance and other factors reported in the survey. We used a linear regression to determine the relationship between bird abundance and total herd size or number of cows in production, controlling for region (Eastern WA vs Western WA) and applying interaction terms for abundance and region (Eq 1). We also analyzed the relationship between bird abundance and milk fat content. We used logistic regression to evaluate the relationship between bird abundance and self-reported presence of Johne’s disease and *Salmonella*. We collapsed the response categories for somatic cell count (SCC) and milk protein content into two (≤ 200,000 and > 200,000 for SCC, and ≤ 3.25% and > 3.25% for milk protein content) and performed logistic regression. Odds ratios and 95% confidence intervals were calculated for significant estimates. All regressions were performed using R, version 3.4.1 [13].

$$Herd\;size=\propto +\beta_{1}⋆Region+\beta_{2}⋆Abundance_{1}+\beta_{3}⋆Abundance_{2}+\beta_{4}⋆Region⋆Abundance_{1}+\beta_{5}⋆Region⋆Abundance_{2}+\epsilon ;\;where\;Abundance_{1}\;is\;1,000\;to\;10,000\;birds\;and\;Abundance_{2}\;is>10,000\;birds.$$ (1)

#### Estimating bird damage

We asked dairy operators reporting bird damage whether feed offered to milk cows becomes contaminated with bird feces, and to estimate the percentage of feed ruined and discarded due to bird damage. We also asked operators if they have observed birds consuming feed, and if so, what percentage of total feed was consumed by birds. We combined consumption and contamination estimates to determine the total amount of feed lost due to the presence of birds. We asked respondents to rank bird abundance, feed consumption, and fecal contamination by season (quarter) from most (1) to least (4). We presented seven bird management methods to operators and asked them to identify any methods they used, their effectiveness (not, somewhat, or very), and cost of these methods.

#### Dairy production and costs

The survey solicited information on herd size, milk production, butterfat and protein content, presence of *Salmonella* and Johne’s disease within the herd, and feed and veterinary costs. Dairy operations test SCC, butterfat, and protein on each tankload of milk sold, and are also paid quality premiums related to these data; thus operators that completed this survey had this information readily available. Survey questions asked dairy operators whether *Salmonella* or Johne’s disease was detected on their dairy in 2016, eliciting a yes/no response. Dairy operators have each tankload of milk tested for overall bacteria, and also specifically test any animals with symptoms of a *Salmonella* infection or Johne’s disease. In particular, Johne’s disease is closely monitored on Washington dairy operations because of its severity. Therefore, information about the presence of *Salmonella* or Johne’s disease within a herd in 2016 was easily accessible to the dairy operators that completed the survey. We examined relationships between bird abundance and herd size, butterfat and protein content, and herd health via regression analysis. Respondents provided estimated veterinary and feed costs for 2016, as well as an estimate of the percentage of feed lost due to bird consumption and spoilage resulting from bird contamination. Dairy operators typically have these estimated costs readily available. Average feed cost was multiplied by the estimated percentage of feed lost due to bird presence, then divided by average herd size to determine cost of feed loss per cow (Eq 2).

$$Cost\;of\;feed\;loss\;per\;cow=\frac{Average\;feed\;cost⋆Percent\;feed\;lost}{Average\;herd\;size}$$ (2)

**Fig 1:**
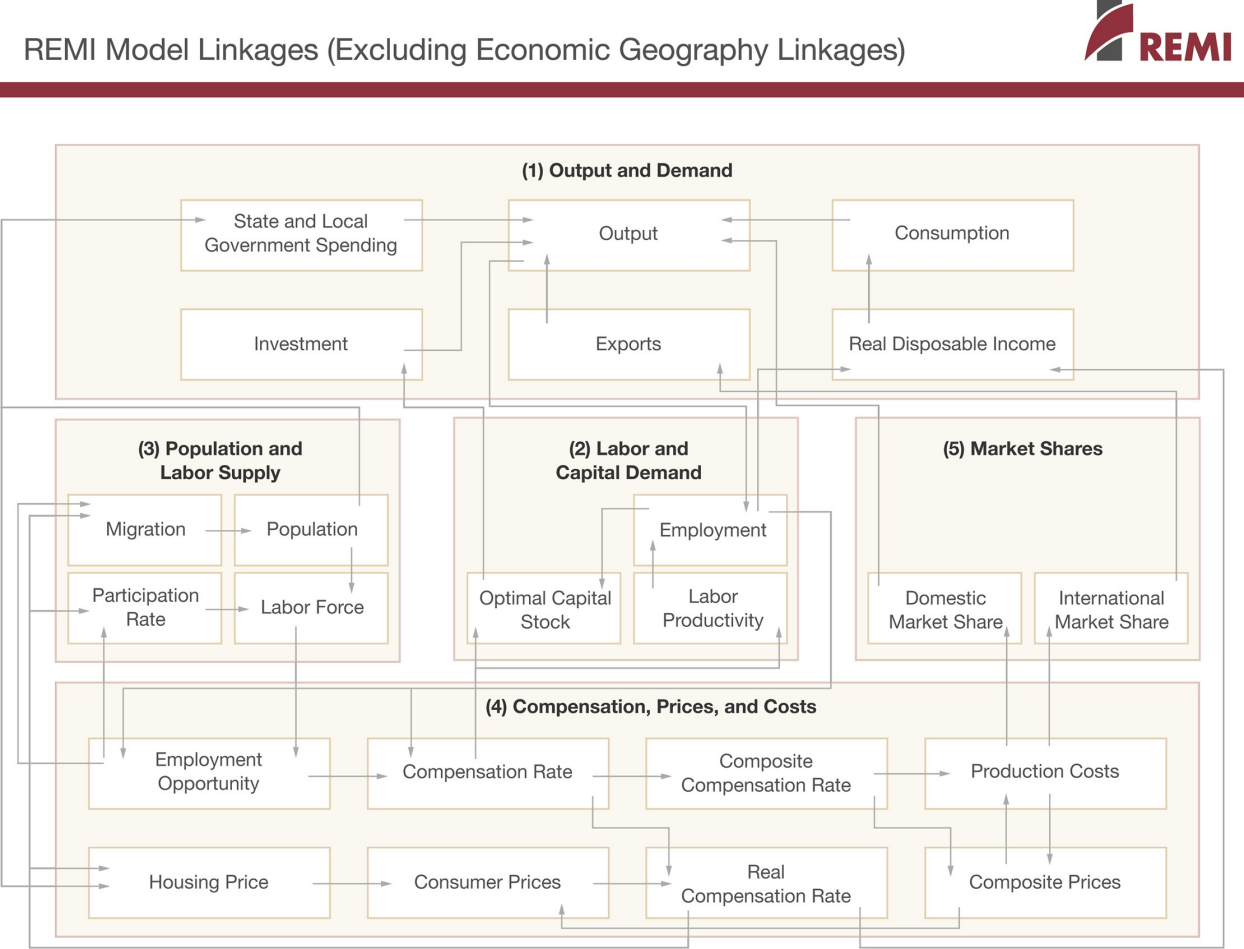
The REMI structural economic forecasting model.

Direct costs of bird presence on dairies (feed lost due to consumption and spoilage) were used to determine the broader impact of bird presence on the local economy using REMI PI+ from Regional Economic Models, Inc. Regional models can illustrate how an impact to the agricultural sector affects people not directly involved in agriculture. REMI is a computer-based simulation model of the U.S. economy that allows modeling at both the national and sub-national scales. This structural economic forecasting model uses a non-survey based input-output (I-O) table, which models linkages among industries and households of a regional economy (Fig 1). The REMI model generates forecasts detailing behavioral responses to changes in price, production, and other economic factors [14]. In other words, REMI can model impacts that changes in the agricultural sector might have on other sectors of the economy and predict changes in employment and income in those sectors. For example, a decrease in feed loss translates to increased income for dairy operators, and may result in increased spending at local restaurants and retail shops, which in turn generates jobs at those businesses. This increased income among workers then translates into further spending. Capturing these ripple effects, or multiplier effects, is vital to understanding the total impact a change in one sector has on the entire regional economy [15].

**Fig 2:**
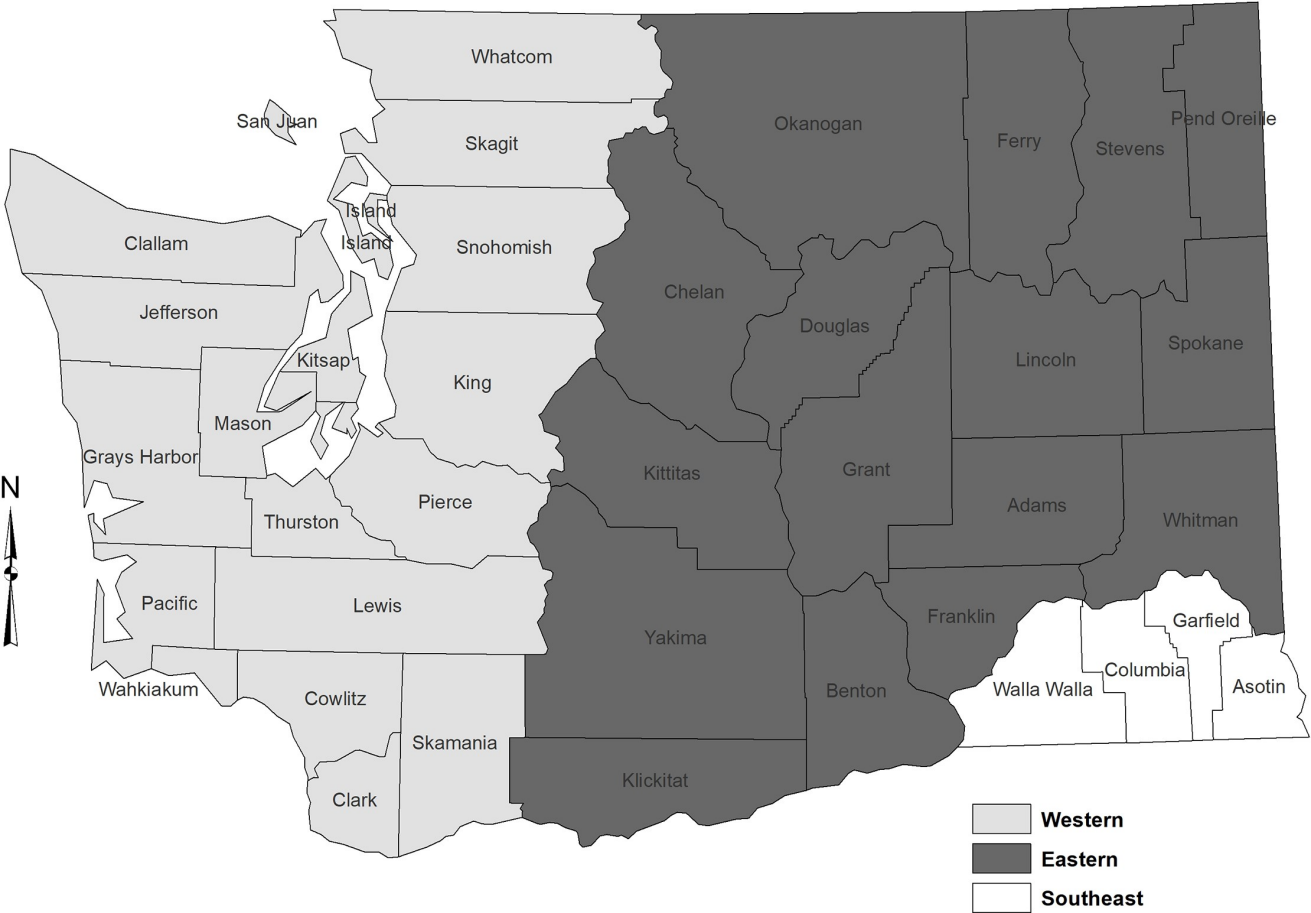
Map of the study area. The southeast region was excluded from the study because it does not contain any dairy operations.

Washington State was divided into two regions, Western and Eastern, excluding the Southeast region of the state (Walla Walla, Columbia, Garfield, and Asotin counties) because there are no dairies in that area (Fig 2). Regional impacts were modeled for each region.

## Results

### Demographics of dairy operations

A total of 49 surveys were returned at the conference, resulting in a response rate of approximately 50%. One survey was a duplicate (two individuals had each filled out a survey for the same farm) and was thrown out. Survey responses were equally distributed between the Western and Eastern regions of WA.

Respondents reported total herd size and number of cows currently in milk production. Herd size varied depending on region, with larger herds found on the Eastern side of the state (Table 1). On average, 77% of cows were currently being milked (range: 25% to 100%). The majority (87%) of dairy operations used free stall housing (N = 47). Some respondents chose more than one type of housing. Those who selected “other” and wrote in “open lot” or “dry lot” (15%) were all located in the Eastern region. Nine percent wrote in “pasture.”

**Table 1:** Number of responding dairy farms by herd size across the state and by region (West and East).

Herd size	State	West	East
<500	12	10	2
500–1000	12	8	4
>1000	24	6	18
Total	48	24	24

### Milk production and quality

**Fig 3:**
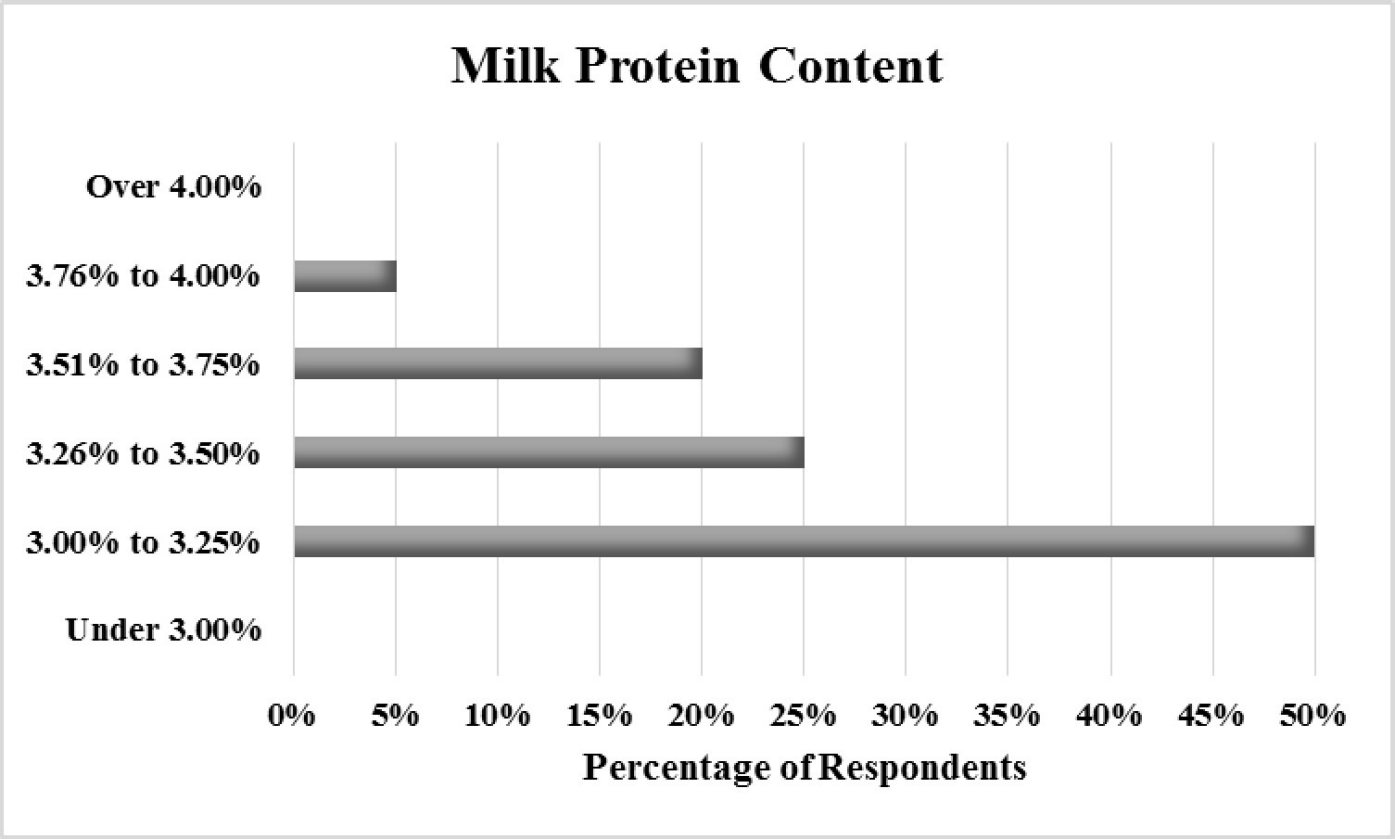
Percentage of respondents reporting each level of protein content.

**Fig 4:**
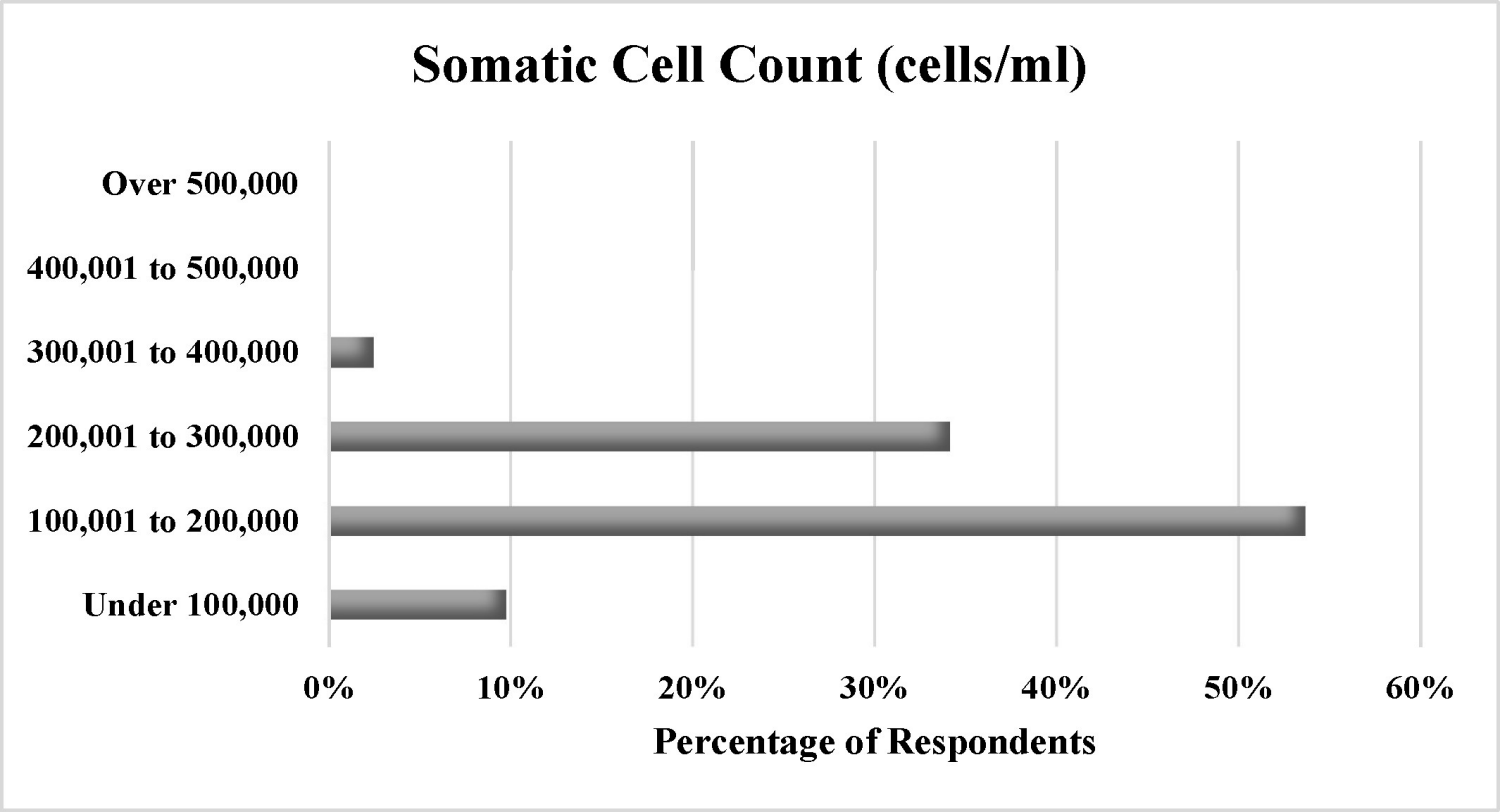
Percentage of respondents reporting each level of somatic cell count.

Survey respondents reported a mean milk butterfat content of 4.1% (range: 3.6 to 5.6%, sd = 0.44). Half of dairy operators indicated a milk protein content between 3.00 and 3.25%. No operators reported protein content greater than 4% or less than 3% (Fig 3.) Over half (54%) of respondents reported SCCs between 100,001 and 200,000 (Fig 4). No operators reported SCCs higher than 400,000.

Just over a quarter of operators reported possible or confirmed Johne’s disease (26%) or *Salmonella* (28%) in their herd. Six percent of operators reported only Johne’s disease, 9% reported only *Salmonella*, 19% reported both diseases, and 66% reported neither disease. Two thirds of positive responses for Johne’s disease and 69% of positive responses for *Salmonella* came from the Eastern region of the state.

### Bird abundance, identification, and management

#### Abundance

**Fig 5:**
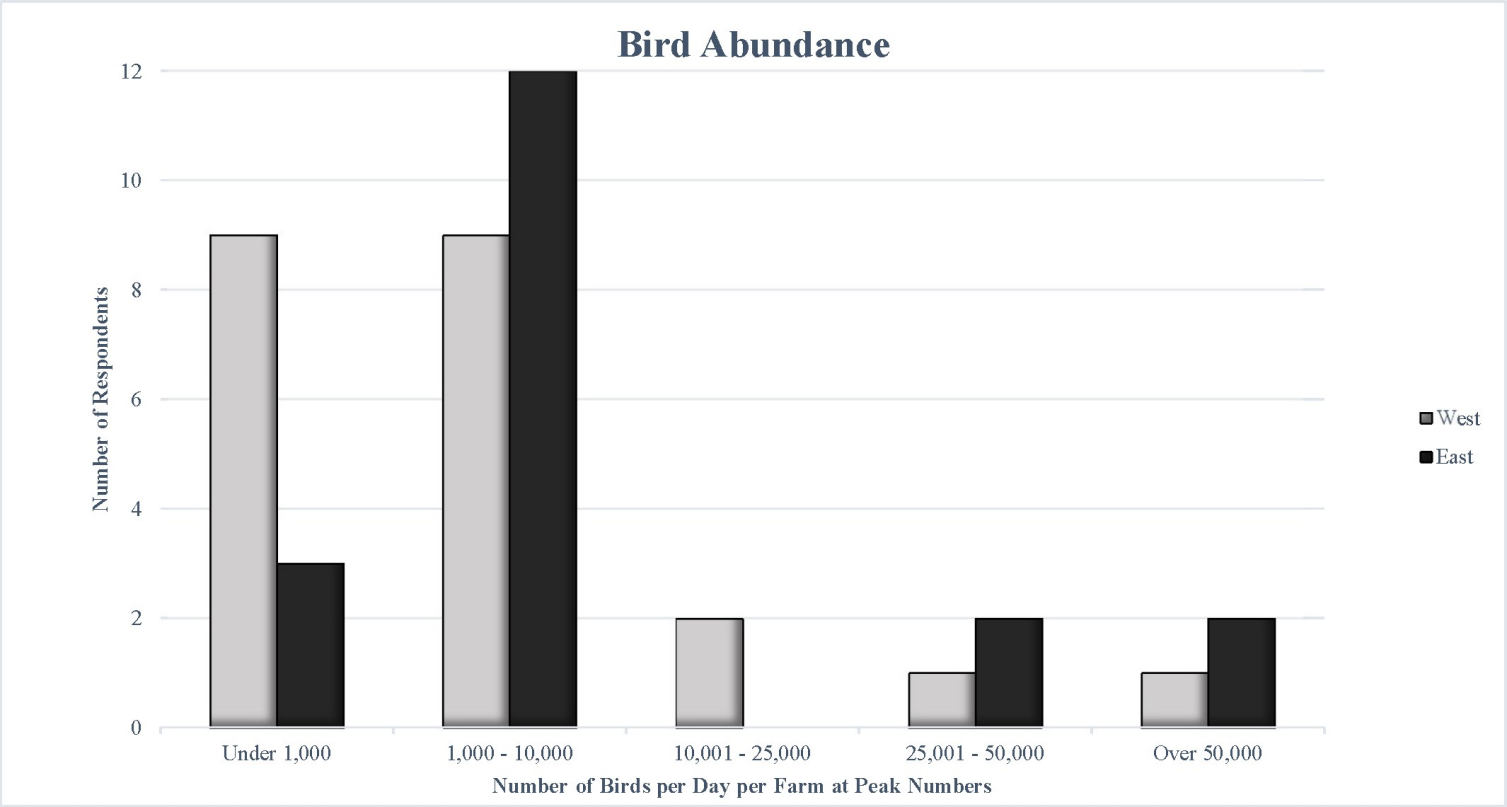
Number of birds present per day on each operation at peak numbers.

Dairy operators provided estimates of the number of birds present on their operations per day at peak numbers in 2016 by selecting from a range of options. The majority (81%) of operators statewide reported seeing 10,000 birds or fewer on their farms per day (Fig 5). Dairy operators in the Western region were evenly split between “Under 1,000” birds and “1,000 to 10,000” birds, while Eastern operators were more likely to report “1,000 to 10,000” birds. Statewide, 51% of operators reported “1,000 to 10,000” birds on their operations per day.

Regression analyses revealed positive relationships between bird abundance greater than 10,000 birds and herd size (Table 2) and self-reported presence of Johne’s disease (OR = 8.334, 95% CI = 1.0343, 67.1491) and *Salmonella* (OR = 77.015, 95% CI = 4.1115, 1442.597). Bird abundance between 1,000 and 10,000 birds was not related to herd size or disease presence. Bird abundance was not related to milk butterfat, protein content, or SCC.

**Table 2:** Relationship between herd size/cows in production and bird abundance, region, and their interaction.

	Total Herd Size				Number of Cows in Production			
	Estimate	Std Error	t-value	p-value	Estimate	Std Error	t-value	p-value
Intercept	451.7	629.1	0.718	0.4777	353.3	472.2	0.748	0.45942
Eastern Region	1448.3	1258.1	1.151	0.2577	853.3	944.3	0.904	0.37255
1,000 to 10,000	190.0	889.6	0.214	0.8322	171.7	667.8	0.257	0.79867
Over 10,000	2617.1	1134.1	2.308	0.0272	2346.7	851.2	2.757	0.00932
East*1,000 to 10,000	1261.8	1517.4	0.832	0.4114	667.5	1138.9	0.586	0.56170
East*Over 10,000	1057.9	1834.0	0.577	0.5679	1046.7	1376.6	0.760	0.45230

Reference case is under 1,000 birds in the Western region. Shaded variable (Over 10,000 birds) is significant.

#### Species identification

Statewide, dairy operators who ranked species identified European starlings as the species most commonly observed consuming feed (85% of operators) and causing the most damage (68% of operators), followed by pigeons. Operators in the Eastern region ranked sparrows in third place, while Western operators identified crows in third place (Table 3).

**Table 3:** Bird species ranked by feed consumption and overall damage to operation.

Birds observed consuming feed				Birds causing most damage to operation			
West		East		West		East	
Species	Score	Species	Score	Species	Score	Species	Score
European Starling	40	European Starling	32	European Starling	47	European Starling	44
Pigeon	28	Pigeon	14	Pigeon	31	Pigeon	34
Crow	9	Sparrow	12	Crow	14	Sparrow	9

#### Bird management

**Fig 6:**
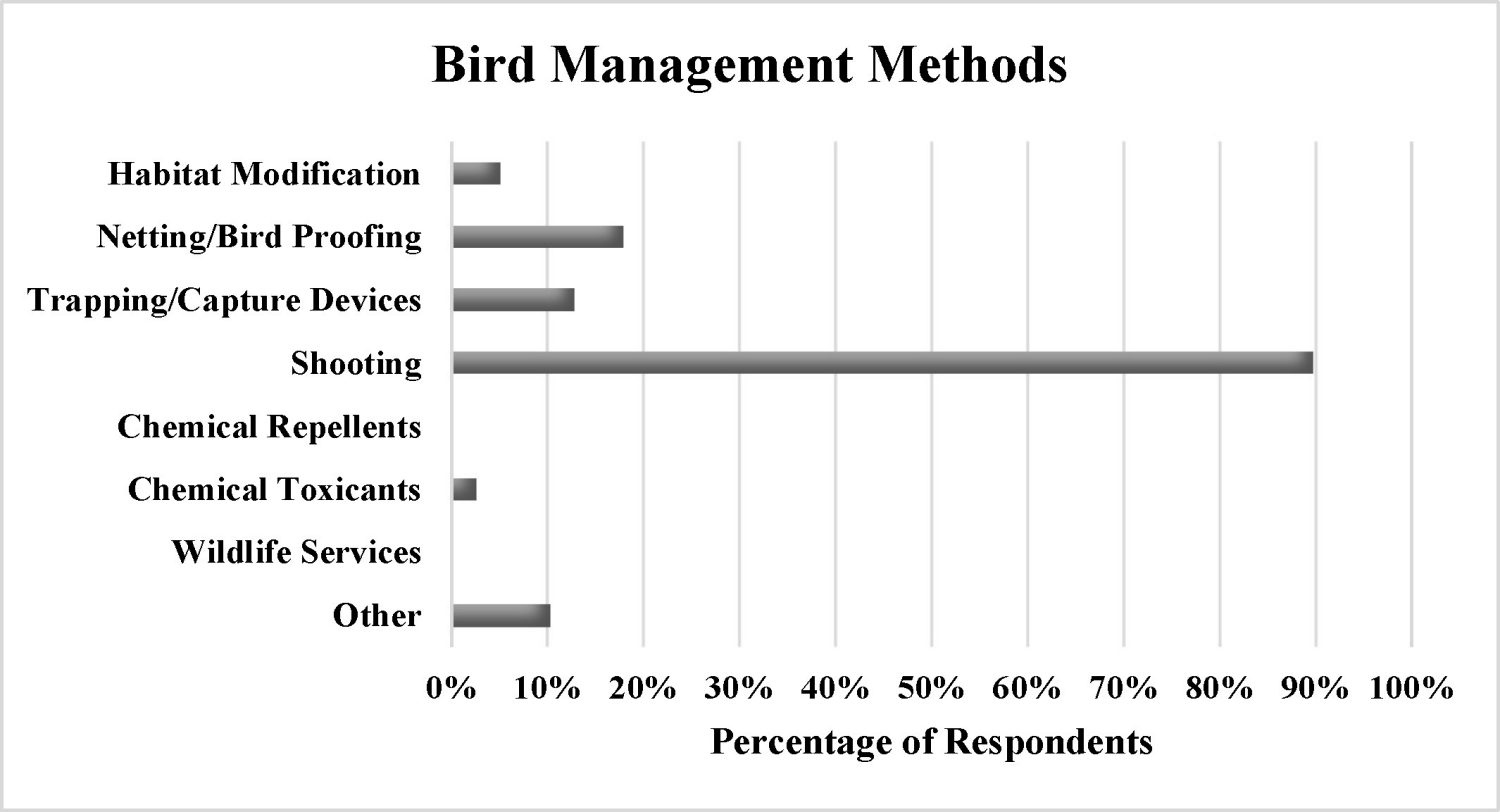
Percentage of respondents using each bird management method.

Most dairy operators (90%) who responded to the question about bird management efforts reported using shooting as a bird management method (Fig 6), and 88% described this method as “somewhat” effective. Respondents spent an average of $555 annually on shooting (median = $100), with several operators indicating shooting is free. Other methods used in 2016 were netting/bird proofing (18%), trapping/capture devices (13%), habitat modification (5%), and chemical toxicants (3%). No respondents reported using chemical repellants or Wildlife Services to manage birds in 2016. Three respondents (8%) wrote in presence of predator birds (hawks or falcons) on their operations and one (3%) wrote in use of predator calls.

In a separate question, we asked dairy operators whether they knew of the work done by USDA/APHIS/Wildlife Services to manage bird damage, and whether they had Wildlife Services manage birds on their dairy between 2014 and 2016. About one third (35%) of operators knew about Wildlife Services, and 12% reported they had Wildlife Services manage birds on their dairy sometime during that timeframe.

### Dairy production and costs

Dairy operators reported losing an average of 4.4% of their feed to birds, split almost evenly between spoilage and consumption (2.64% and 2.87% respectively). The sum of spoilage and consumption estimates is greater than the average because some operators only reported one of the two estimates, so the other was assumed to be zero. Therefore, this study’s loss estimate is likely conservative. Using this estimate, we calculated feed loss of $54.55 per cow per year or $54,550 per year for a farm with 1,000 cows. In 2016, the average milk income in Washington State was $3,809.26 per cow [16, 17]. Feed loss from birds alone equates to a 1.43% decrease in revenue per cow. Using a three year average of the number of cows in each county [18], we calculated the value of feed lost to dairy operators in the Western and Eastern regions of Washington. The Western region is estimated to lose about $5.5 million per year, and the Eastern region loses about $9.2 million per year.

We used the above estimates as inputs in REMI, modeled as lost dairy operator income. Regional economic modeling predicted decreased farmer income resulting from feed loss due to birds will result in a net loss of 73 jobs and $6.7 million in gross domestic product (GDP) over five years in the Western region of the state, and a net loss of 81 jobs and $5.9 million in GDP over five years in the Eastern region of the state (Table 4).

**Table 4:** Five-year regional and statewide impacts of feed lost due to birds.

	West	East	State
**GDP**	$6.7 million	$5.9 million	$12.6 million
**Jobs**	73	81	154

## Discussion

Throughout this survey, dairy operators in Washington State indicated bird presence on their dairies is an important health and economic concern, associating birds with increased disease presence and reporting significant feed loss. Dairy operators also suggested they lack effective options for managing birds, with the vast majority using shooting and few using other techniques. This is consistent with a pilot study on dairies in British Columbia that found birds habituated quickly to auditory deterrents such as predator call recordings and propane cannons [19].

Although a few respondents noted presence of predator birds at their dairies, none indicated the use of professional bird abatement falconry or installation of nest boxes or perches to attract raptors. A growing body of literature suggests the use of predator birds may be an effective method of deterring pest birds. Falconry has been successfully used to reduce the number of pest birds in leafy greens fields [20], in blueberry fields [21], and in strawberry fields [22]; some of these studies indicate falconry may be most effective in combination with visual deterrents. Steensma et al. [19] found hawk kites to be potentially effective visual deterrents on dairies. Shave et al. [23] found installation of predator nest boxes at fruit orchards was a cost-effective method of attracting kestrels and reducing pest bird numbers. These methods could be further explored to evaluate their efficacy on dairies.

Three of the four bird species most frequently implicated in causing damage are invasive species in North America (European starlings, rock pigeons, and house sparrows), and their impacts as described in this study are an example of the need for effective management of invasive species. It is possible that starlings, in particular, find a highly attractive year-round habitat in the region due to proximity of fruit crops through summer and fall, and dairy feeds in open commodity sheds, free-stall barns, and feedlot dairies year-round. Their migration and flocking patterns, and persistence in overwintering in dairy barns, are challenging for both fruit and dairy farms [24]. Likely a combination of methods will be the most effective at providing long term persistent and cost-effective management of pest birds. Exclusion netting, while expensive, has been shown to be fairly effective and when paired with the inexpensive method of installation of nest boxes and perches to attract raptors may be a viable and practical management option.

Due to the small sample size of this study, some research questions could not be answered. For example, many respondents did not report milk production and some of those who did reported numbers in unclear units and timeframes. As a result, we did not have enough data to examine the relationship between bird abundance or pathogen presence and milk production. However, this study provides information that allows producers to not only understand the costs and benefits of their management methods but also conveys the macroeconomic implications of reducing damage from pest birds. The direct costs of feed loss alone due to birds was $14.7 million for the state of Washington, comprising over one percent of total milk production value in the state [25]. This does not include lost revenue resulting from increased pathogen incidence or potentially increased cattle stress [26] due to pest bird presence on operations, making this a conservative estimate. With the volatility of milk prices and tight proft margins on dairies in the U.S., even a small change in the cost of production can affect an operation’s economic sustainability.

Engaging the public by illustrating the community-based benefits of pest bird management, providing education regarding the problems associated with pest birds, and encouraging potential “natural” solutions such as falconry and native predator habitat enhancement, could allow for a broader understanding and acceptance of these management practices.
